# The High PMNs Phagocytosis Resistance of Enterococcal Isolates from RTx Patients 

**DOI:** 10.1155/2015/432579

**Published:** 2015-03-10

**Authors:** Tomasz Jarzembowski, Agnieszka Daca, Jacek M. Witkowski, Ewa Bryl, Bolesław Rutkowski

**Affiliations:** ^1^Department of Microbiology, Medical University of Gdańsk, Do Studzienki 38 Street, 80-227 Gdańsk, Poland; ^2^Department of Pathology and Experimental Rheumatology, Medical University of Gdańsk, Dębinki 7 Street, 80-211 Gdańsk, Poland; ^3^Department of Pathophysiology, Medical University of Gdańsk, Dębinki 7 Street, 80-211 Gdańsk, Poland; ^4^Department of Nephrology, Transplantology and Internal Medicine, Medical University of Gdańsk, Dębinki 7 Street, 80-952 Gdańsk, Poland

## Abstract

Infections caused by opportunistic pathogens such as enterococci remain difficult to manage, especially in immunocompromised patients. Because of infections' limited symptoms in such patients the additional problems are to find proper diagnostic criteria and the management of infection. Here we aimed to compare the resistance of commensal enterococcal strains and RTx patients' isolates, to PMNs phagocytosis. Thirty-six enterococcal urine and faecal isolates from RTx patients and 17 faecal isolates from healthy volunteers were cultured in planktonic and biofilm forms in 37°C or 42°C. Another tested variable was the addition of immunosuppressant to the culture media. Bacterial cells were stained with fluorescent reporter (CFDA, PI) and incubated with PMNs. Results of phagocytosis were estimated as a mean fluorescence intensity (MFI) of PMNs using flow cytometry. Commensal enterococci cultured in all abovementioned (37°C and 42°C/the addition of immunosuppressant) conditions were less resistant to phagocytosis compared to RTx isolates. Observed significant difference in phagocytosis resistance suggests that patients in immunosuppression are colonized with high risk strains which may lead to the development of infection.

## 1. Introduction

Phagocytosis is an essential process to initiate immunity to bacterial infection so the resistance to this mechanism may be a key property resulting in opportunistic infection. Phagocytes combine the role of innate immune effectors and acquired immune responses. They initiate a microbial death pathway by routing ingested pathogens to lysosomes and also by targeting the phagocyte oxidase complex to the phagolysosome. Additionally, phagocytic leukocytes, particularly dendritic cells (DC), utilize phagocytosis to direct antigens to both MHC I and MHC II compartments [1]. Role of neutrophils includes defense against many common microorganisms and they are essential for the control of common bacterial infections. An example of increasing importance is enterococcal infections [2, 3]. Genus* Enterococcus*, usually regarded as a normal commensal of intestinal tract, is increasingly considered as a cause of nosocomial infections. In the last decade, enterococci have been reported as the second most common cause of wound and urinary tract infection and the third most common cause of bacteremia [4, 5]. It is assumed that many enterococcal infections are endogenous, originating from the intestinal tract [6]. Wells et al. speculated that macrophages may serve as a vehicle facilitating translocation from the intestine into the lymph system and bloodstream.

The risk of enterococcal infection is especially high in patients undergoing immunosuppressive therapy due to, for example, organ transplantation. Among hospitalized patients, renal transplant (RTx) recipients are at a high risk of developing infectious complications, caused not only by established pathogens but also by commensal bacteria [7, 8]. In the early phase after renal transplantation, when patients are exposed to the most intense immunosuppression and enterococcal infections are most common, even life-threatening infections can present with mild or virtually no clinical symptoms.

Our previous study showed the significant differences in gene expression and biofilm properties [9, 10] between enterococcal isolates from RTx patients and healthy volunteers. Here, phagocytosis resistance to PMNs was analyzed.

## 2. Material and Method 

### 2.1. Bacterial Culture

Thirty-six enterococcal urine and faecal isolates were isolated from RTx patients hospitalized at the Medical University of Gdańsk. Full characteristic of patients is presented in Table 1. All patients initially underwent induction with monoclonal (basiliximab) or polyclonal antibodies (ATG) and were prescribed subsequently TAC (tacrolimus) + MMF (mycophenolatemofetil)/MPS (mycophenolate sodium) + glucocorticosteroids or CsA (cyclosporine) + MMF/MPS + glucocorticosteroids or CsA + everolimus + glucocorticosteroids.

As a reference group, 17 enterococcal strains of* Enterococcus faecalis* were isolated from healthy volunteers from Gdańsk region. The isolates were identified to species level by strep ID test (BioMerieux) and classified as different strains of* Enterococcus faecalis* by biochemical and resistance profiles. All bacterial strains were stored at (−70°C) in brain heart infusion (BHI) broth with 25% (vol/vol) glycerol. Biofilms of these strains were obtained by microtiter method in 36 h culture at 37°C on flat-bottom wells (TRP, Switzerland). The cells were then removed by sonication and resuspended in 0.9% NaCl. The planktonic cells were cultured in BHI at 37°C and 42°C; the culture at 37°C was carried with or without the addition of 2000 ng/mL of tacrolimus for 24 h.

#### 2.1.1. PMNs Isolation and Phagocytosis Assay

PMNs were isolated from EDTA-anticoagulated venous blood of normal healthy adult volunteers by automated magnetic isolation (MiltenyiBiotec, autoMACS Pro, Whole Blood CD15 MicroBeads, human). For each phagocytosis assay, enterococci from an overnight culture in BHI broth were diluted to 1 : 100 in fresh BHI broth and grown to mid-log phase. After washing in NaCl, the bacterial density was adjusted spectrophotometrically to a concentration of 2 × 10^9^ CFU/mL. Enterococci were then stained with propidium iodide (PI) at room temperature. Each phagocytic mixture contained 0.4 mL of stained enterococci (2 × 10^8^/mL) and 0.4 mL of neutrophils (2 × 10^4^) was incubated for 45 min at 37°C. To remove adherent but not phagocyted bacteria, 0,4 mL of trypsin was added and incubated for another 10 min at 37°C.

Fluorescence of particles was determined using a FACScan flow cytometer (Becton-Dickinson, Franklin Lakes, NJ, USA). The fluorescence of neutrophils resulting from phagocyted bacteria (FL3) was normalized by fluorescence of bacterial strain used in the experiment. As a control, stained culture bacteria and suspension of neutrophils without bacteria were used. Results were tested by analysis of variance (ANOVA) by StatSoft software (Statistica 10, USA).

## 3. Result 

**Figure 1:**
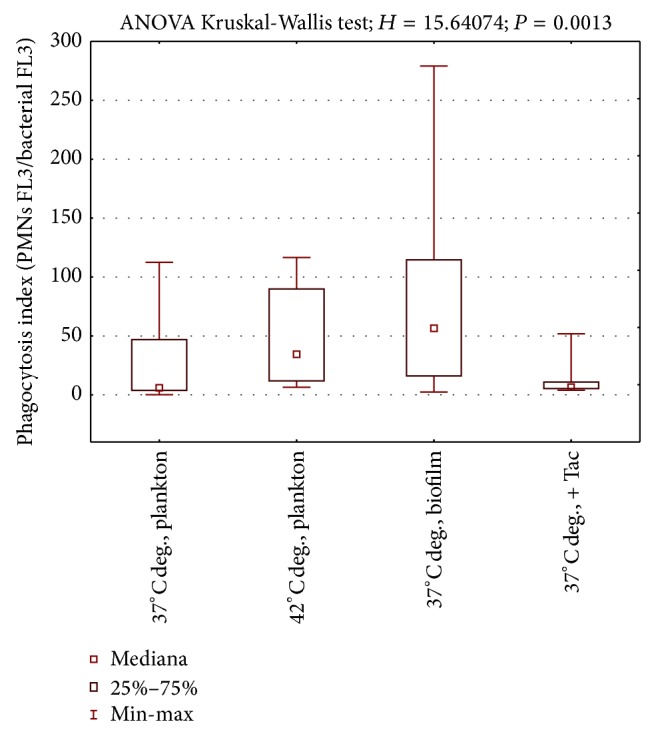
Phagocytosis of commensal strains. 37°C deg., planktonic culture at 37 Celsius degrees; 42°C deg., planktonic culture at 42 Celsius degrees; 37°C deg. + Tac, planktonic culture at 37 Celsius degrees with addition of tacrolimus; 37°C deg. biofilm form, cells isolated from biofilm cultured at 37 Celsius degrees.

**Figure 2:**
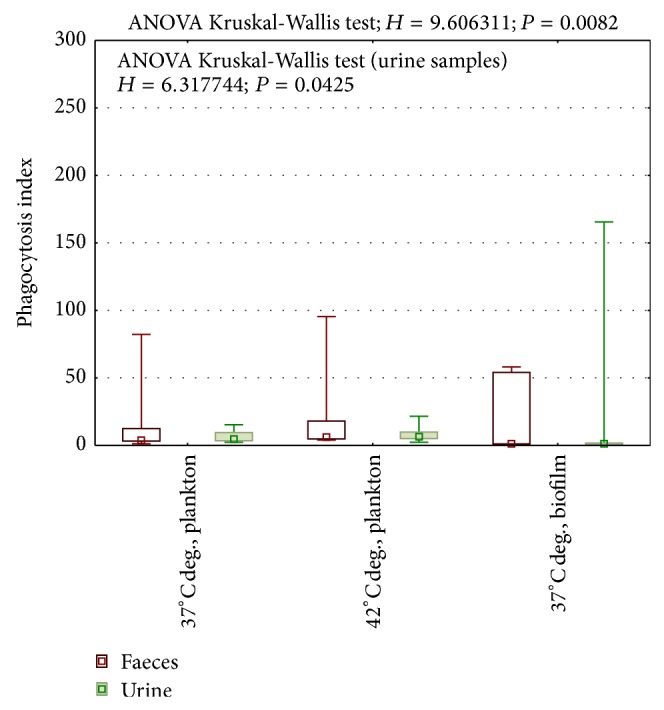
Phagocytosis of medical strains. 37°C deg., planktonic culture at 37 Celsius degrees; 42°C deg., planktonic culture at 42 Celsius degrees; 37°C deg. biofilm form, cells isolated from biofilm cultured at 37 Celsius degrees.

When enterococci were cultured at 37°C, phagocytosis index (FL3/FL3 of bacteria) varied from 1,1 to 112 in commensal strains and 1,2 to 82,2 in medical strains. What is significant is that commensal strains presented higher phagocytosis index than RTx isolates at 37°C (Mann-Whitney *U* test; *z* = 2,02; *P* = 0,04) in all studied culture conditions (Figures 1 and 2). Both higher temperature and biofilm form of culture significantly increased phagocytosis index in this group of enterococci (Figure 1).

In contrast to RTx isolates, cells of commensal strains released from biofilm were higher in susceptibility to phagocytosis than planktonic cells (Wilcoxon test *z* = 2,2; *P* = 0,025). At 42°C commensal strains were not only more sensitive to phagocytosis than at 37°C (Wilcoxon test *z* = 2,8 *P* = 0,005), but also more sensitive than medical strains at the same conditions (Mann-Whitney *U* test; *z* = 2,82; *P* = 0,0047). The difference between commensal and medical strains was also proved in biofilm forming cells (Mann-Whitney *U* test; *z* = 3,725; *P* = 0,0002).

While RTx faecal samples reaction presented some similarity to the commensal faecal samples, opposite variation was noticed in urine isolates from RTx patients (Figure 2, Kruskal-Wallis test). The difference between phagocytosis index at 37°C and 42°C was definitely lower (geometric means 4,27 and 6,61, resp., Figure 2).

## 4. Discussion

Many factors are believed to determine the virulence of* Enterococcus* species. They include adherence properties of the strain and survival in the phagocytes. For example, the aggregation substance (Agg) has been shown to form large aggregates and hence may contribute to pathogenesis. It is thought that another enterococcal protein, ESP, promotes adhesion, colonization, and evasion of the immune system and plays some role in antibiotic resistance. A group of hydrolytic enzymes including hyaluronidases, gelatinase, and serine protease are also probably involved in the virulence of* Enterococcus* species, although their precise roles are yet to be clearly understood [4, 5].

Despite the fact that many virulence traits have been described, no significant progress in diagnostic has been reached. Furthermore, some results suggest that individual factors may not be crucial for enterococcal pathogenicity [11]. For example, the widespread distribution of putative virulence determinants in the faecal baby isolates supports the conception of enterococcal virulence, not as a result of any single virulence factor but as a more complex process [11].

Resistance of enterococci to PMNs phagocytosis was also described many years ago [12], but the reason of such phenomenon remains unclear. It was proved that presence of AS protein inhibits phagocytosis of bacteria by macrophages [2]. Others also noticed, for example, that the presence of aggregation protein [3] or EPA gene [13] protects enterococci from being killed by PMNs. In contrast to other reports, we found no correlation between AS gene expression and resistance of strains to phagocytosis by PMNs (data not shown). Because of high diversity of strains, no general difference in resistance to phagocytosis can be proved. However, detailed analysis brings evidences of specific properties of the strains. First of all, susceptibility to phagocytosis increases when temperature increases to 42°C in both commensal and clinical isolates. It was previously indicated that temperature elevation is critical for survival from pathogens [14]. It is generally accepted that temperature elevation acts to enhance the host immune response rather than pathogen growth or survival. However, the way in which temperature elevation translates into increased resistance to infection is poorly understood [14]. Results of our study suggest that the changes in bacterial properties may by one of such mechanism.

According to expectation, in medical strains, cells released from biofilm were more resistant to phagocytosis than planktonic cells (FL3 1,24 to 4,27, resp.). However, high susceptibility to phagocytosis of biofilm cells of commensal strain (median FL3 60,31) is surprising and may explain how high biofilm forming enterococci colonizing gastrointestinal tract [15] may be balanced in intestine by immune system in healthy people. On the other hand, here we failed to prove the existence of the differences in phagocytosis resistance between faecal and urine isolates from RTx patients so the environment conditions do not seem to be the only factor of high resistance of RTx isolates to phagocytosis.

Primary immunosuppression for kidney transplant recipients includes cyclosporine and tacrolimus. The second line of the drugs from chemical point of view belongs to macrolides, the same group as antibacterial agents. In fact, their influence on expression of bacterial proteins was already described [9]. In the current study, to evaluate the influence of tacrolimus on phagocytosis resistance, the drug was additionally added to the culture broth of commensal strain. Despite the fact that such effect was observed (Figure 1) direct influence of tacrolimus on bacterial cells was not proved.

Many authors suggest endogenous nature of enterococcal infection. Olivier and others [16] suggest that the most colonized patients who develop VRE BSI are infected with their own colonizing strains. Using PFGE for comparison, Shay et al. [17] found that, amongst 11 paired stools and blood VRE isolates, 8 were identical. Similarly, Montecalvo et al. [18] found closely related stool and blood VRE isolates in 3 patients during an outbreak in an oncology ward. On the other hand, in 2006, Leavis and others [19] proposed identification of high risk clonal complex of* E. faecium*. Also our results support hypothesis that enterococcal strains' specific phenotypic profile rather than immunotherapy makes enterococcal isolates more resistant to phagocytosis.

Although we found our observation significant, it is disappointing that high diversity of phagocytosis resistance limits possibility of application of the results as a diagnostic marker. Temporary lack of symptoms in volunteers colonized by virulent strains may be a reason of such situation. Thus, study on animal model is required to evaluate the method.

## Figures and Tables

**Table 1:** Characteristic of patients.

	All	Hypertensive nephropathy	Systemic vasculitis	PKD^*^ + ADPKD^**^	Glomerulopathy	Wilms' tumor (1) and unspecified causes (2)
*n*	19	2	6	5	3	1 + 2
Age	51,63 ± 16,49	66 ± 1,41	49,67 ± 15,56	51,2 ± 14,55	60 ± 8,66	38,33 ± 27,43
Sex (W/M)	12/7	0/2	4/2	5/0	2/1	1/2
Time after transplantation (years)	0,84 ± 0,91	1,25 ± 0,35	0,67 ± 0,52	0,6 ± 0,55	2 ± 1,73	0,17 ± 0,29
UTIs episodes (present/absent)	3/16	0/2	0/6	0/5	2/1	1/2
UTIs number	0–6	1/4	0	0	1/5/6	6/0/0
CMV infection (present/absent)	4/15	2/0	1/5	0/5	0/3	1/2
CRP	13,39 ± 29,60	16,40 ± 1,98	7,90 ± 14,02	4,50 ± 3,90	43,93 ± 71,60	3,70 ± 2,85
Leukocytosis	9,06 ± 3,41	5,91 ± 0,42	8,43 ± 2,97	8,72 ± 2,88	10,11 ± 6,06	11,91 ± 2,05
Leukocyturia (present/absent)	6/13	1/1	1/5	2/3	2/1	0/3

^*^PKD: polycystic kidney disease; ^**^ADPKD: autosomal dominant polycystic kidney disease.
